# Digital localization of intraoperative samples enhances margin analysis precision in oral squamous cell carcinoma

**DOI:** 10.3389/fmed.2025.1638833

**Published:** 2025-09-29

**Authors:** Adam Michcik, Aleksandra Ciarka, Łukasz Garbacewicz, Adam Polcyn, Piotr Choma, Barbara Wojciechowska, Barbara Drogoszewska

**Affiliations:** ^1^Department of Maxillofacial Surgery, Faculty of Medicine, Medical University of Gdańsk, Gdańsk, Poland; ^2^Department of Maxillofacial Surgery, University Clinical Centre in Gdańsk, Gdańsk, Poland; ^3^Department of Pathomorphology, Faculty of Medicine, Medical University of Gdańsk, Gdańsk, Poland; ^4^Department of Clinical Pathomorphology, University Clinical Centre in Gdańsk, Gdańsk, Poland

**Keywords:** frozen section analysis, intraoperative methods, oral cancer, surgical margin, virtual imaging, cancer local control

## Abstract

**Objectives:**

The intraoperative margin assessment technique enhances local control of Oral Squamous Cell Carcinoma (OSCC). However, the standard frozen section analysis (FSA) procedure does not allow precise interpretation of the final histopathological results. For this reason, we have developed a modified intraoperative margins (IM) assessment protocol using virtual 3D images of resected OSCC.

**Material and methods:**

The pilot study included 10 patients with OSCC located in the floor of the mouth (FOM, *n* = 3), tongue (TC, *n* = 2), maxilla (MT, *n* = 1), and retromolar triangle (RMT, *n* = 4). After excision, OSCCs were scanned and mapped, marked on the virtual preparation of the IM collection sites. The modified pathological assessment of the IM enabled the evaluation of dimensions, including the width of the collected IM.

**Results:**

By analyzing virtual OSCC images, we demonstrated the effectiveness of a modified IM assessment methodology. In one case study, the oncological council disqualified the patient from adjuvant radiotherapy.

**Conclusion:**

Leveraging the OSCC’s virtual mapping capabilities and the modified IM pathology methodology, we developed an IM evaluation protocol that enables the incorporation of IM width into the OSCC’s microscopic excision margin, thereby significantly enhancing the diagnostic value and precision of the final OSCC histopathology results.

## 1 Introduction

Oral squamous cell carcinoma (OSCC) is the most common malignant tumor of the oral cavity (1, 2) and holds the sixteenth position in malignancy worldwide (1). OSCC initially develops insidiously, without obvious symptoms, based on a precancerous lesion or *de novo* (3). One of the first symptoms is usually pain (4, 5), followed by dysfunctions depending on the original location. The increasing number of younger patients with OSCC (6) and unsatisfactory treatment outcomes (1, 7) obliges us to search for new therapeutic solutions constantly. Many OSCC prognostic factors are known and described, including DOI – depth of invasion, lymphovascular invasion (LVI), perineural invasion (PNI), and extranodal extension (ENE) (2, 8–10). However, it is still essential to perform OSCC resection with an appropriate size of the histopathological margin. The concept of a clear, close, and involved surgical margin was introduced by the UK Royal College of Pathologists (11). Generally, a histopathological resection margin of at least 5 mm is considered sufficient; narrower margins increase the risk of recurrence (12–14). The collection of intraoperative specimens from the tumor bed constitutes a standard procedure for assessing oncological purity following the excision of the primary specimen. Various methodologies exist for assessing intraoperative margins: FSA frozen section analysis (15), tumor-targeted fluorescence (TTF) techniques (16), touch imprint cytology (TIC) (17), or techniques that employ stains or dyes to exploit structural or metabolic differences between normal and pathological mucosa (18). In many cases, the procedures used do not enable us to accurately utilize the location and size of the IM collected from the main formulation during the subsequent assessment of histopathological margins. After collecting intraoperative margins from the tumor bed and sending them to the laboratory, we lose a significant amount of valuable information regarding their size and exact location, which is especially crucial in the case of long margins from the main specimen and orientation, including any side markings on the IM samples. We provide the pathologist with information regarding the intraoperative margin (IM) collection site. In large specimens with intricate anatomy and extensive margins, accurately identifying the corresponding IM donation site on the primary specimen can prove challenging. Intraoperative margin assessment solely determines the presence of cancer within the slice. The number of positive IMs is influenced by various factors, including the proficiency and experience of the surgical team (19). The accurate assessment of oncological free margins using the standard FSA procedure is often hindered by considerable inaccuracies and the risk of misinterpreting results, particularly with regard to classifying suspicious sites. Acknowledging these deficiencies, we sought a solution that would facilitate the precise identification of both the location and size of the IM on OSCC specimen. We have developed a protocol designed to visualize and archive resected tumors, including mapping, labeling, and measuring the collected intraoperative margins (IM). This approach enables the integration of IMs into assessing the histopathological margin of excision for oral squamous cell carcinoma (OSCC) during the final evaluation following formalin fixation. In narrow histopathological margins, including IM width alongside the histopathological margin, may influence therapeutic decisions, potentially reducing the necessity for reoperation or adjuvant radiotherapy for the patient. Modern imaging techniques, including the creation of virtual 3D images of the excised tumors, offer a wide range of previously unavailable possibilities for imaging and mapping tumors. The straightforward and reproducible protocol we proposed for virtual communication between surgeons and pathologists (20) also creates new opportunities for utilizing intraoperative margins assessment.

This study aimed to establish a protocol to enhance the precision of excision margin assessment for oral squamous cell carcinoma (OSCC) by integrating IM into the surgical margin evaluation. The application of virtual imaging techniques enabled us to accurately delineate the IM donor sites on the primary specimen, thereby allowing us to consider their width as an additional component of the surgical excision margin.

## 2 Materials and methods

The pilot study included 10 patients with oral tumors who underwent surgery in the Maxillofacial Surgery Department of the Medical University of Gdańsk from August to October 2024. All participants signed a written consent. Patients with OSCC T1 to T4b located in the floor of the mouth (FOM, *n* = 3), tongue (TC, *n* = 2), maxilla (MT, *n* = 1), and retromolar triangle (RMT, *n* = 4) were enrolled in this study. All patients were over 18 years of age (mean age: 71.2). Participants in this study were not disqualified based on the presence of general diseases. In the case of qualification for surgical treatment, these general diseases did not impact the procedure for tumor excision. All qualified tumors were excised en bloc, which made it possible to perform a full tumor scan. Fragmentation or removal of the OSCC for technical reasons in pieces disqualified from the study Table 1.

**TABLE 1:** Characteristics margin.of the study group, 8th TNM classification used, legends: TC, tongue cancer; FOM, floor of the mouth; MT, maxilla cancer; RMT, retromolar triangle; TPS, tumor primary site; G, grading; pT, tumor size; DOI, depth of invasion; LIM, length of intraoperative margin; WIM, Width of intraoperative margin; HIM, height of intraoperative margin.

Characteristic		N	%
Gender	Male-	6	60
	Female	4	40
Age	50th	1	10
	60th	3	30
	70th	4	40
	80th	2	20
TPS	TC	2	20
	FOM	3	30
	RMT	4	40
	MT	1	10
G	G1	1	10
	G2	6	60
	G3	3	30
pT	pT1	2	20
	pT2	3	30
	pT3	3	30
	pT4	2	20
DOI	≤5 mm	2	20
	5–10 mm	6	60
	>10 mm	2	20
LIM	10–20 mm	13	26
	20–30 mm	22	44
	30–50 mm	15	30
WIM	≤2 mm	14	28
	3–4 mm	28	56
	>4 mm	8	16
HIM	≤3 mm	16	32
	4–5 mm	19	38
	>5 mm	15	30

### 2.1 Methods

Following the OSCC’s excision, IMs were obtained from the tumor bed (defect-driven intraoperative assessment). It should be noted that, with minor methodological adjustments, the proposed virtual FSA analysis can also be applied to specimen-driven margin assessment. It is a method that has gained increasing support in recent years. However, by using the pioneering method of virtual mapping, we eliminated most of the imperfections associated with defect-driven intraoperative assessment, and for this reason, this method was employed in the described procedure. The standard procedure involved collecting four margins in a clockwise orientation: anterior, medial, posterior, and lateral. In certain instances, a margin was also procured from the base of the tumor bed. The IM collection sites were immediately marked on the main specimen by sewing a bead or inking, which prevented incorrect mapping of the virtual object after the scanning procedure, especially in cases with long margins and anatomically challenging tumor shapes. The IMs were marked to help with their orientation. Two threads were sewn on from the side of the radial IM margin, and one thread was designated as the medial, lateral, or side margin for both the front and rear of the IM. In the second stage, photographic documentation was made on graph paper, and IM samples were measured for archiving.

Due to the small size and difficulties in maintaining the shape, we could not perform precise scans of the IM samples. Tumor scanning: after mating with spray (21) the tumor was placed on the designed stand. Next, scanning was performed using a 3D scanner: Revopoint MINI–Dual-axis Turntable Package (Shenzhen, China), adapted to scan small objects with an accuracy of 0.02 mm at 10 frames per second. The scanner was chosen for its ultra-high resolution and resistance to ambient light (Supplementary Video).

**FIGURE 1:**
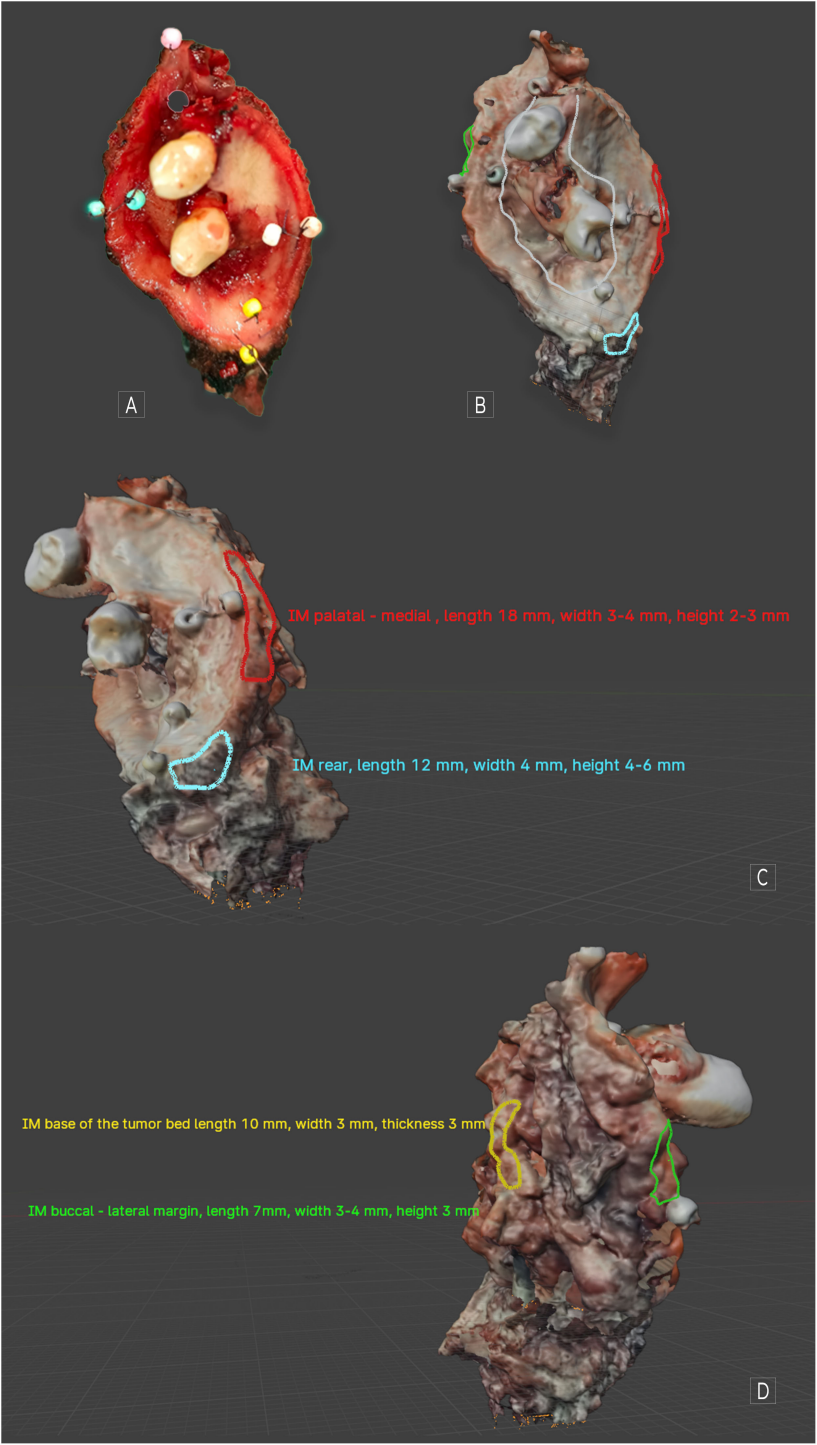
MT cancer. **(A)** Visible image after excision; **(B)** scan of the same tumor covering a part of the hard palate along with the premolars and a fragment of the cheek. Ulceration is marked in white. **(C)** Visible mapped IMs medial and rear. **(D)** Visible mapped IMs: lateral and base of the tumor. When using Blender 4.1.1, we can assess the object spatially. Individual descriptions from the marking have been excluded to increase the readability of the figures. Applying any markings directly on the object’s surface and descriptions around it is possible.

Upon creating a virtual image of the excised OSCC, the tumor was mapped utilizing Blender version 4.1.1 (open-source software^1^). On the surface of the virtual tumor, we accurately indicated the specific locations considering their dimensions, corresponding to the sites from which IM was extracted from the tumor bed. This mapping occurred immediately following the collection of the samples, thereby facilitating the identification of corresponding areas on the OSCC scan without any complications Figure 1.

In the mapped virtual OSCCs, the dimensions of the margins (length, width, height) were described in detail, providing the pathologist with comprehensive information about the parts of the main margin that represented the IM and its width. Typically, both the medial and lateral margins were extended, sometimes matching the length of the histopathological margin. The uneven surface of the main specimen does not affect the ability to map the tumor (due to the possibility of virtual drawing directly on its surface, regardless of its shape). In this case, during pathological assessment, the main margin is divided into more fields, each corresponding to individual H&E slides of the main specimen. With the ability to accurately identify the locations of H&E slides and the positions and sizes of IMs, we can sum their microscopic details widths. This approach ensured that all identified invasive margins for a given tumor were marked and documented. Once finalized, the file was uploaded to the cloud, where the pathologist could download it for use during the histopathological assessment of the main specimen. Then, the collected IM were immediately sent to the laboratory for intraoperative evaluation.

### 2.2 Intraoperative assessment

**FIGURE 2:**
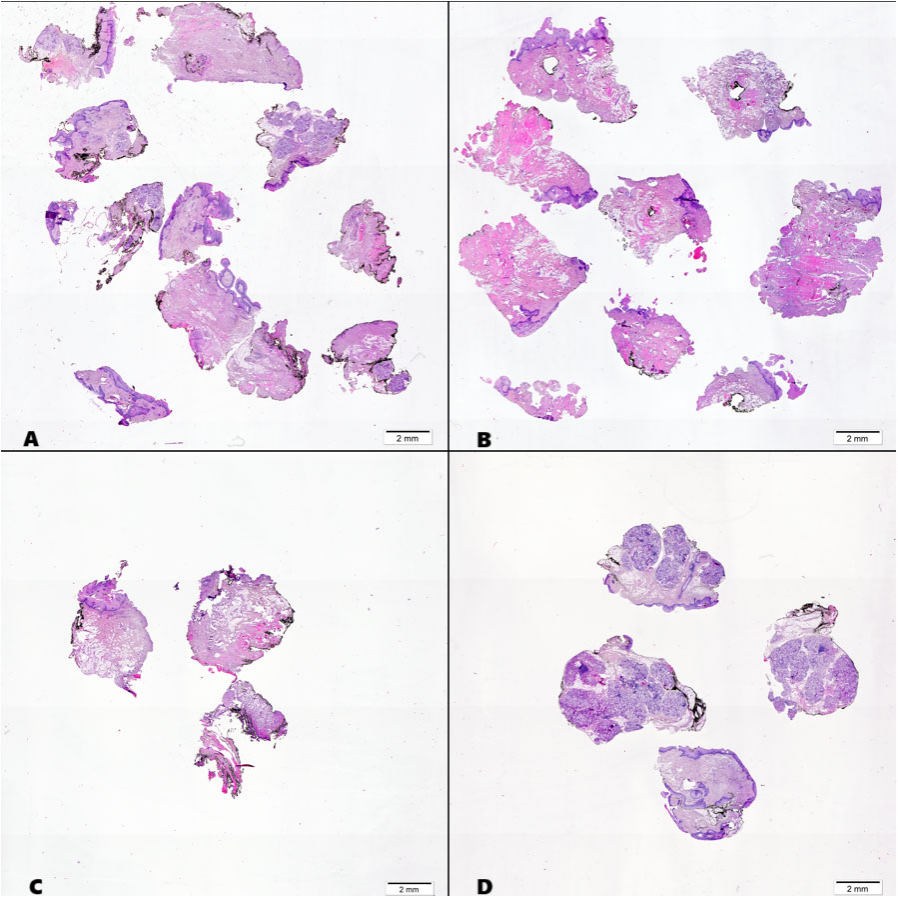
Histological sections of intraoperative margins from the oral cavity, stained with hematoxylin and eosin (H&E): **(A)** Medial FOM (floor of the mouth) margin; **(B)** Lateral Buccal margin; **(C)** Posterior margin; **(D)** Anterior margin.

Surgically excised margins, previously marked with identifying sutures, were submitted for intraoperative frozen section analysis. Each margin specimen was placed in a separate, appropriately labeled container to maintain specimen integrity and prevent misidentification, which is crucial for determining the appropriate extent of surgical excision should malignancy be detected. Upon receipt, the pathologist conducted a comprehensive gross examination of each specimen. The margins were then sectioned perpendicularly to the resection plane into uniform slices measuring 3–5 mm in thickness. These tissue sections were carefully oriented and mounted on labeled specimen holders, ensuring that the cut surfaces remained exposed and accessible for microscopic evaluation. The prepared tissue specimens were rapidly frozen at −23 °C using standard cryostat protocols. Serial sections of 3–5 μm thickness were obtained using a cryostat microtome under controlled temperature conditions. The resulting cryosections were immediately processed using standard hematoxylin and eosin (H&E) staining protocols. Microscopic examination was performed using an Olympus BX43 light microscope equipped with objective lenses providing magnifications ranging from 2× to 40×. Each section was systematically evaluated to assess margin status and identify any residual neoplastic tissue at the resection boundaries Figure 2.

## 3 Results

The proposed OSCC virtual image protocol significantly improved the accuracy of histopathological margin assessment. By incorporating the IM width into the evaluation of histopathological margins, a more precise determination of the proper OSCC cut margins was achieved.

Visualizing the IM collection sites, particularly those with a narrow margin on the virtual oral squamous cell carcinoma (OSCC) specimen, presents novel opportunities for histopathological evaluation. This advancement enables pathologists to provide a more precise and detailed analysis of the specimen preparation, thereby facilitating clinicians in making informed and accurate postoperative therapeutic decisions.

Ten patients with OSCC have been successfully enrolled in our pilot study. In multidisciplinary meetings involving the oncology team, comprising maxillofacial surgeons, oncologists, and radiologists, therapeutic decisions were modified for one patient. This adjustment occurred through the utilization of virtual tumor imaging along with comprehensive histopathological evaluations.

**FIGURE 3:**
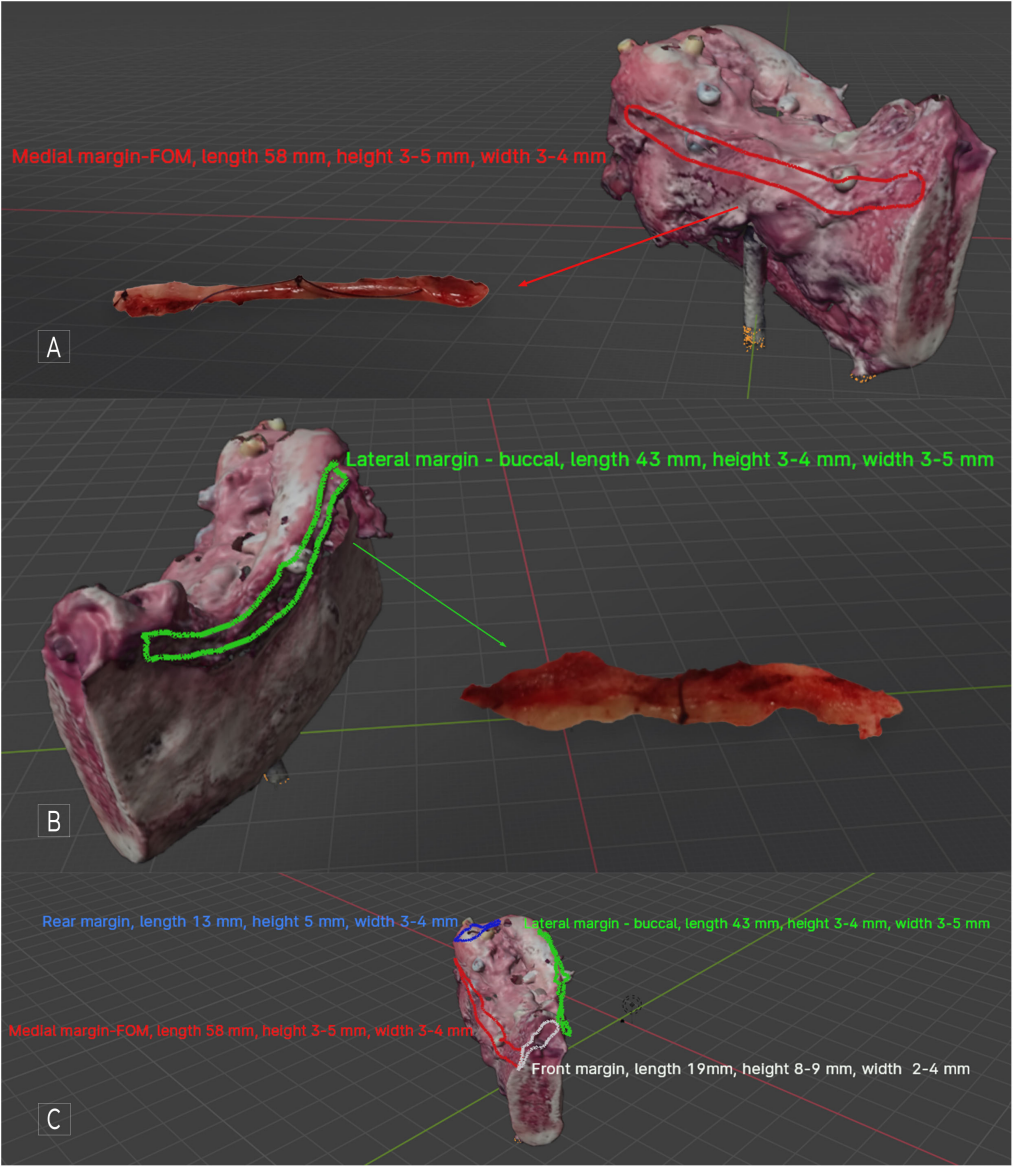
Patient disqualified from adjuvant radiotherapy after analysis of histopathological result, including IM width excision in microscopic margins. RTM OSCC with segmental mandible resection - tumor scan, visible, marked, and described IM; **(A)** medial side view, with the mapped IM FOM visible along the entire length FOM margin; **(B)** lateral side view, with the buccal IM mapped and described visible along the entire length of the margin; **(C)** all mapped IMs and their descriptions are visible. With software, it is possible to switch individual markings and descriptions on and off to increase the object’s transparency. In Blender, previewing the virtual specimen from all sides and angles is possible.

In this case, OSCC RMT (G1, LVI-, PNI-, DOI 4 mm, WPOI – 1. Cut-off lines free from cancer, metastasis-free lymph nodes 0/18, margins amounted to: front and rear = 5 mm, FOM and buccal microscopic margin = 2,5 mm (pT2N0 - TNM Classification of Malignant Tumors, 8th Edition), simultaneous reconstruction with free fibula flap (FFF). This result qualified the patient for adjuvant radiotherapy. During the final evaluation of the result and the confrontation of the histopathological result with the virtual mapped image of the OSCC, it was found that IM FOM was: length 58 mm, height 3–5 mm, and width 3–4 mm, IM buccal was: length 43 mm, height 3–4 mm, width 3–5 mm Figure 3.

The methodology used for histopathological assessment of IM allowed for the accurate determination of the IM specimen’s width along its entire length, as well as determining the exact location of the individual H&E slides. This made it possible to sum the microscopic values of the main specimen’s FOM and buccal margins with IM FOM and IM buccal. As a result, the margins originally described as R1 (2.5 mm) turned out to be R0 (5.5–7 mm), allowing adjuvant radiotherapy to be avoided and the patient to qualify for observation only. Of course, the IMs taken are usually shorter than the corresponding margin of the main specimen. However, even in this case, the virtual mapping capabilities of the OSCC allow us to precisely determine the location and size of the IM, which makes it possible to determine in which section of the major margin we can include the width of the IM in the microscopic resection margin.

## 4 Discussion

The preservation of an appropriate excision margin in OSCC is paramount and represents a critical prognostic factor (22–24). In clinical practice, a margin of ≥5 mm in the microscopic resection of OSCC is generally considered standard. This threshold is crucial for ensuring adequate tumor clearance and minimizing the risk of local recurrence (25–27), however, there are analyses indicating different cut-offs (28, 29). Determining safe resection margins that reduce local recurrence rates and the risk of nodal metastasis is difficult. It must be analyzed in conjunction with other factors such as Pattern of Invasion, differentiation, Lymphovascular Invasion (LVI), Perineural Invasion (PNI) (30). Especially due to the diverse Pattern of Invasion, including aggressive, dispersed tumor invasion patterns, margins <5 mm predict worse survival (31, 32). Considering the abovementioned factors, maintaining a microscopic margin of at least 5 mm is warranted (26). When planning a tumor excision, it is crucial to consider the impact of formalin fixation (FF) on the histopathological evaluation and the final interpretation of results. The standard procedure for FF used to preserve excised OSCCs affects the size of the MRM. The study conducted by our team showed an unequivocal shrinkage of MRM. Based on 947 measurements of virtual OSCC scans, we showed MRM shrinkage of up to 28% (*p* val. = 5.06 × 10-76) (33). Interestingly, our findings did not reveal a significant effect of FF on the tumor itself, which contrasts with the results reported in other studies (34).

The intraoperative examination technique is used not only in head and neck cancers (35–38), and among those available, the most used is Frozen Section Analysis (39). Due to the limitations of this technique, researchers are continually exploring alternative solutions. Mohs micrographic surgery is one of the alternative proposed methods. It is characterized by high precision and accuracy and it is typically utilized by dermatologists. Although accurate, it has limitations in its use in oral cancers (40). Gauthier et al. proposed the use of this method in tongue cancers (41). However, for other OSCC locations, its application appears technically infeasible. Use of fluorescence techniques (42, 43), marking margins with toluidine blue (44, 45) or spectroscopy (46–48) in the evaluation of intraoperative margins serves as an alternative to FSA. The Near-Infrared-Labeled Cetuximab and Panitumumab for Optical Imaging technique was proposed by Day et al. He demonstrated the high effectiveness of this method in the identification of residual disease, which, in the opinion of the researcher, allows this method to be used as an alternative to intraoperative OSCC assessment (49). Spectroscopy, a type of focused analysis, could be significant in the intraoperative evaluation of resection boundaries in oral cancer. Unlike histological methods, spectroscopy provides a non-invasive and immediate way to assess margins during surgery. Aaboubout et al. proposed the use of Raman spectroscopy to assess the excision margins of OSCC. Their study achieved a high specificity of 98% and a sensitivity of 78%. According to the researchers, these promising results suggest that Raman spectroscopy can effectively evaluate the margins of OSCC, similar to other existing methods (50). The potential to digitally archive Raman spectroscopy images opens up new possibilities for its application in the method our team developed for creating virtual images of OSCC. In this approach, digital Raman spectroscopy images could be uploaded and overlaid at corresponding points on a virtual model of the resected OSCC. This presents an exciting alternative to the FSA procedure and could lead to further research directions, especially with the use of OSCC virtual archiving techniques. Zanoni et al., using a confocal microscope to assess the OSCC boundaries, demonstrated the high effectiveness of this method. Reflectance confocal microscopy demonstrates high sensitivity and positive predictive values for tumor identification (83.3%). This method necessitates costly equipment and the expertise of a skilled physician for proper evaluation (51). Considering its sensitivity and specificity, as well as its non-invasive nature, this method is an intriguing approach to assessing the margins of OSCC. The optical imaging methods used to assess the excision boundaries in OSCC allow for the evaluation of excision margins directly in the operating room without the involvement of a pathologist. It is important to note that this responsibility falls entirely on the surgical team. Consequently, the outcomes will rely heavily on the operator’s experience and subjective judgment. Dynamic optical contrast imaging (DOCI) is an imaging technology developed by Tajudeen et al. The fundamental contrast technique used by DOCI is the same as that applied in fluorescence lifetime imaging, where the natural lifespan of fluorophores in tissue is analyzed using a pulsed, long-wave UV light source for illumination. Tajudeen suggested using DOCI during surgery, but specifically on a previously prepared sample in the pathology laboratory. He found this method to be highly effective in distinguishing between cancerous and healthy tissue. Consequently, he recommended DOCI as a viable option for intraoperative examination (52). Intraoperative frozen section analysis (FSA) remains the predominant method for intraoperative tissue evaluation. Specimens are obtained from the tumor bed or the primary specimen following resection to ascertain whether the tumor has been resected in its entirety. The techniques employed for the collection of FSA slices may differ. Samples are taken according to the scheme (53), or in places selected by the operator as the most suspicious. Interestingly, among all surgical specialties, the evaluation of resection margins during surgery using the frozen section technique is most commonly conducted for cancers of the head and neck (54). Intra-operational margin assessment increases local control but does not affect regional control (24, 55, 56). Enhancing on-site control supports its implementation, but the FSA standard method has several limitations. It is important to note that most of these limitations have been addressed in our protocol. A significant challenge associated with the utilization of FSA is accurately identifying the specific locations within the tumor bed from which the samples were procured in instances of a positive diagnosis. A methodology proposing a solution to this problem was suggested by Aaboubout et al., who used sewn-in markers surrounding the tumor (57). This method may not be universally effective for all cases of OSCC, particularly in cases involving highly infiltrative tumors, where it may not be possible to secure the marker with sutures. In our methodology, once a virtual representation of the tumor has been generated, we are capable of mapping the tumor to any location that corresponds with the taken FSA sample. The specific location from which a sample is obtained–whether from the periphery, the bottom of the tumor bed, or the tumor itself–holds no significant bearing on the outcome. Moreover, the capability to archive and retrieve images during surgical procedures or reoperations significantly enhances the surgeon’s precision in locating these areas. Research indicates that surgeons have shifted the FSA resample collection sites by about 1 cm (58). Olsin et al. conducted a study using 3,758 FSA samples, proposing a three-level method to minimize the risk of sampling error (59). The suggested solution is rather complicated. In our protocol, in the case of larger specimens, we additionally marked them with threads to increase the amount of information provided to the pathologist intraoperatively. The determination of the radial margin of the section and its side allowed for the correct interpretation of the sample during FSA procedure. The standard procedure for FSA involves taking samples directly from the tumor. Some researchers suggest that sampling should only be performed on specimens. Chang et al. conclude that taking samples from the tumor bed may increase the risk of local recurrence in cases of tongue cancer (60). Collecting specimens from a tumor bed using the standard FSA procedure, particularly for large tumors, poses a high risk of error without a precise assessment of the tumor’s size and location. This lack of accuracy can hinder the correct interpretation of the final results. Our method’s ability to map the excised oral squamous cell carcinoma (OSCC) facilitates accurate interpretation of intraoperative results in addition to postoperative histopathological findings. However, it is important to note that some studies suggest the advantages of obtaining FSA from the tumor bed following a standard protocol (24). It is associated with a significant potential for inaccuracies, particularly in cases involving extensive tumors with long margins. This risk arises when the surgeon obtains only a small fragment of the margin, which constitutes merely a fraction of the whole. Similar conclusions were drawn by Ribeiro et al. in their research findings (61). In this situation, without being able to pinpoint the exact location of the sample, it is impossible to accurately interpret the results and identify the area of concern. The methodology outlined in this paper enables a thorough assessment of the intraoperative margin width, something that cannot be achieved with standard procedures (62). The authors emphasize that accurately mapping the resection margins and the sites for intraoperative sample collection is crucial. This accuracy allows the width of the intraoperative margin to be considered when determining the microscopic excision margin for OSCC. This consideration is vital, as it can significantly impact the status of the microscopic margin in the OSCC excision. The proposed method does have some drawbacks. It requires a trained team to operate a 3D scanner and use software for processing 3D objects. The surgical procedure may experience a slight increase in duration due to the time required for scanning and mapping the tumor, as well as identifying the IM collection sites. Furthermore, adjustments to the FSA evaluation technique by the pathologist can contribute to the overall lengthening of the procedure. This can lead to extended waiting times for the surgical team to obtain feedback from the pathology laboratory. The scanning process may not always yield a comprehensive image of the tumor, as certain areas, particularly deep undercuts or recesses, can remain obscured from the scanner’s view. Nevertheless, the advantages offered by our method significantly surpass these limitations during surgical procedures. With the continuous advancements in 3D technology, it is anticipated that this methodology will evolve further, thereby enhancing its simplicity and potential integration with augmented reality.

In conclusion, we introduced a novel method for using intraoperative examination to evaluate the microscopic margins of OSCC excisions more accurately. The ability to create virtual images of the excised OSCC has opened up new possibilities for assessing and archiving tumors that were previously unavailable. In our pilot study involving 10 patients, we demonstrated the effectiveness of our method and the legitimacy of its use. The ability to determine the exact location of the sample collection for intraoperative examination and its size, including width, significantly increases the accuracy of the OSCC intraoperative examination procedure. We are full of optimism that our method has a chance to become one of those adopted in broadly understood surgical practice.

## Data Availability

The original contributions presented in this study are included in this article/Supplementary material, further inquiries can be directed to the corresponding author.

## Footnotes

1
https://www.blender.org/
